# The novel antidiabetic medications on diabetic retinopathy: relevant molecular mechanisms, advancing diagnostic innovations, and therapeutic implications

**DOI:** 10.3389/fmed.2025.1670643

**Published:** 2026-01-13

**Authors:** Song Wen, Chenglin Xu, Yue Yuan, Lijiao Chen, Yishu Ren, Zhimin Xu, Jianlan Jin, Jiyu Li, Ligang Zhou

**Affiliations:** 1 Pudong Medical Center, Department of Endocrinology, Shanghai Pudong Hospital, Fudan University, Shanghai, China; 2 Fudan Zhangjiang Institute, Fudan University, Shanghai, China; 3 Pudong Medical Center, Department of Surgery, Shanghai Pudong Hospital, Fudan University, Shanghai, China

**Keywords:** artificial intelligence (AI) diagnostics, diabetic retinopathy, incretin-based therapy, optical coherence tomography (OCT), SGLT-2 inhibitors, type 1 diabetes, type 2 diabetes

## Abstract

Diabetic retinopathy (DR), a major cause of vision loss in working-age adults, manifests as a microvascular complication of diabetes, with early-stage non-proliferative diabetic retinopathy (NPDR) requiring timely intervention. This review explores the molecular mechanisms underlying early DR, including microvascular damage, inflammation, oxidative stress, and advanced glycation end products, with distinct profiles in type 1 and type 2 diabetes. Novel antidiabetic medications, such as GLP-1 receptor agonists, SGLT-2 inhibitors, and dual GIP/GLP-1 agonists, target these pathways, may have potential to reduce NPDR progression expected in clinical trials. Advanced diagnostics, including ultra-widefield fundus photography, OCT, OCTA, and AI-based algorithms, achieve over 95% accuracy in detecting NPDR and predicting systemic risks like cardiovascular disease. This article highlights the therapeutic implications of novel antidiabetic drugs, advocating for integrated diagnostic and treatment strategies to mitigate DR's global burden and preserve vision.

## 1 Introduction

Diabetic retinopathy (DR), a leading cause of vision impairment among working-age adults, affects approximately 146 million individuals globally, with projections estimating an increase to 180 million by 2030, driven by the rising prevalence of diabetes, particularly in low- and middle-income countries (1). This microvascular complication of type 1 diabetes mellitus (T1DM) and type 2 diabetes mellitus (T2DM) progresses through distinct stages, from non-proliferative diabetic retinopathy (NPDR) to proliferative diabetic retinopathy (PDR) and diabetic macular edema (DME). Early-stage NPDR, characterized by microaneurysms, capillary dropout, and subtle vascular changes, is often asymptomatic, making it insidious and prone to progressing undetected until irreversible retinal damage occurs (2). As such, NPDR represents a critical window for early intervention to prevent vision-threatening complications.

The pathophysiology of early DR involves a complex interplay of hyperglycemia-induced microvascular damage, inflammation, oxidative stress, and advanced glycation end product (AGE) accumulation (3). These mechanisms disrupt the blood-retinal barrier (BRB), leading to vascular permeability and retinal ischemia. Notably, T1DM and T2DM exhibit distinct profiles: T1DM is marked by rapid hyperglycemic stress and acute vasculitis, while T2DM is compounded by chronic inflammation and systemic comorbidities like obesity, hypertension, and dyslipidemia. Despite the β-cell loss in both diseases, the core pathophysiology of these two conditions may differ. T1DM is characterized by an abrupt insulin deficiency that triggers intense hyperglycemic excursions and an acute vasculitis-like inflammatory response with rapid leukocyte adhesion (4–6). At the same time, T2DM is dominated by chronic low-grade inflammation amplified by insulin resistance and metabolic syndrome (4–6). Recent studies highlight the retina's role as a non-invasive biomarker for systemic complications, with retinal vascular changes—such as arteriolar narrowing and venular dilation—correlating with cardiovascular disease, stroke, and chronic kidney disease (CKD) (7, 8). For instance, the Atherosclerosis Risk in Communities (ARIC) study reported that retinal microvascular abnormalities predict a 15% higher risk of heart failure in diabetic patients, underscoring the retina's prognostic value (9).

Advances in diagnostic technologies, including ultra-widefield fundus photography, optical coherence tomography (OCT), OCT angiography (OCTA), and artificial intelligence (AI)-based algorithms, have revolutionized early NPDR detection, achieving over 95% accuracy and enabling prediction of systemic risks (10). Therapeutically, established interventions like glycemic control and lifestyle modifications are complemented by novel pharmacotherapies, such as finerenone, GLP-1 receptor agonists, SGLT-2 inhibitors, and dual GIP/GLP-1 agonists, which reduce NPDR progression by 8%−15% in clinical trials (11–13). Emerging approaches, including neuroprotective agents, anti-inflammatory therapies, and gene-based interventions, further expand treatment options (14–16). Real-world evidence supports these strategies, with low-AGE diets and structured exercise programs reducing NPDR incidence by up to 10% (17, 18).

This review synthesizes the molecular basis of early-stage DR, compares its pathophysiology between T1DM and T2DM, evaluates diagnostic innovations, and explores therapeutic advancements supported by recent clinical trials and real-world data. It specifically contrasts the molecular differences between T1DM and T2DM in early NPDR, integrating the latest clinical and mechanistic evidence. By integrating insights from ophthalmology, endocrinology, and cardiovascular medicine, we aim to guide clinical practice and research to reduce the global burden of DR, preserve vision, and enhance quality of life for diabetic patients.

## 2 Pathophysiology: molecular and cellular mechanisms of early-stage DR

**Figure 1:**
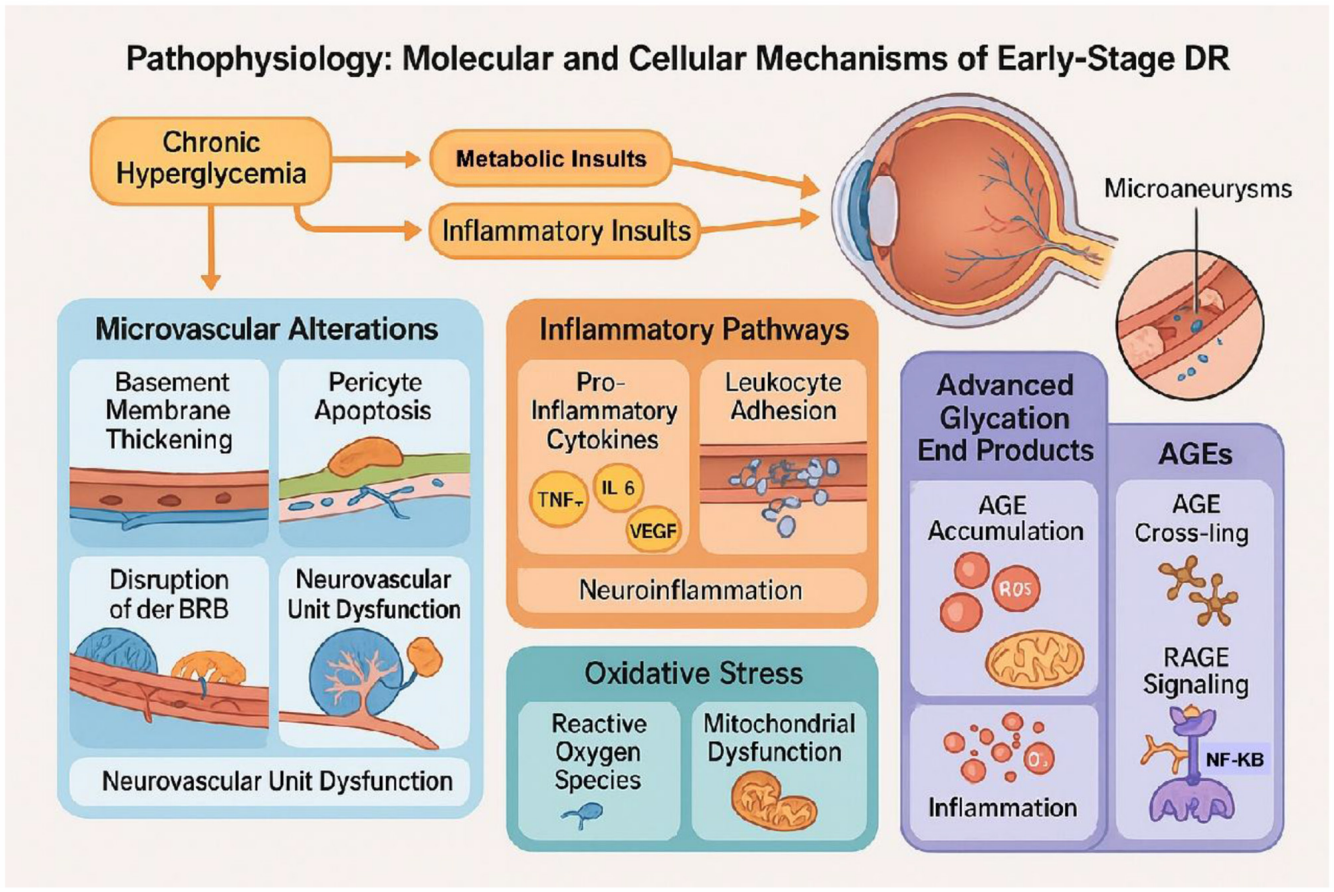
Molecular pathways of early diabetic retinopathy. This figure illustrates the key molecular pathways contributing to early diabetic retinopathy (DR), including hyperglycemia-induced microvascular damage, inflammation, oxidative stress, and advanced glycation end product (AGE) accumulation.

The pathogenesis of early-stage DR involves a cascade of metabolic, inflammatory, and vascular insults triggered by chronic hyperglycemia (Figure 1).

### 2.1 Microvascular alterations

Chronic hyperglycemia, the hallmark of diabetes mellitus, induces profound structural and functional changes in the retinal microvasculature, setting the stage for early-stage diabetic retinopathy (DR). These changes include basement membrane thickening, pericyte apoptosis, endothelial dysfunction, and subsequent disruption of the blood-retinal barrier (BRB), which collectively contribute to increased vascular permeability and the formation of microaneurysms, the earliest clinically detectable lesions in non-proliferative diabetic retinopathy (NPDR).

#### 2.1.1 Basement membrane thickening

This alteration in basement membrane results from excessive extracellular matrix deposition, driven by hyperglycemia-induced upregulation of transforming growth factor-beta (TGF-β) and fibronectin. This process impairs nutrient diffusion and weakens vascular integrity. Study has proven that TGF-β signaling in retinal endothelial cells is amplified in early DR, promoting collagen cross-linking and basement membrane rigidity (19). Basement membrane thickening is the earliest irreversible structural sign of non-proliferative diabetic retinopathy (NPDR), developing within months of hyperglycemia and serving as the strongest histological indicator of progression to vision-threatening stages. It hampers nutrient and oxygen diffusion, decreases capillary flexibility, disrupts pericyte-endothelial communication, traps vascular cells leading to apoptosis, and promotes a self-reinforcing cycle of extracellular matrix buildup driven by TGF-β/CTGF (20, 21).

#### 2.1.2 Pericyte apoptosis

Pericyte apoptosis is universally recognized as the earliest functionally significant and morphologically identifiable lesion in diabetic retinopathy, occurring months to years before microaneurysms become visible clinically. It also serves as the strongest independent histological predictor of progression from no-DR or mild NPDR to moderate/severe NPDR and eventual proliferative disease (22). This early event compromises the structural support of retinal capillaries. Pericytes, which regulate capillary tone and maintain BRB integrity, are particularly vulnerable to hyperglycemia-induced oxidative stress. Crosstalk between pericytes and endothelial cells is compromised in NPDR, mediated by reduced platelet-derived growth factor (PDGF) signaling (23). Specifically, diminished PDGF-BB/PDGFR-β interactions lead to pericyte loss, destabilizing capillaries and promoting microaneurysm formation (24). The molecular change associated with pericyte apoptosis involves hyperglycemia-driven oxidative stress, AGE-RAGE signaling, and disrupted PDGF-B/PDGFR-β survival crosstalk, which contributes to the loss of capillary tone regulation, blood-retinal barrier breakdown, acellular capillary formation, and irreversible ischemia (22, 25). Recent single-cell RNA sequencing data further elucidated that pericyte subpopulations are selectively depleted in early DR, correlating with increased capillary dysfunction (25).

#### 2.1.3 Endothelial dysfunction

Endothelial dysfunction is a critical early factor in NPDR, the initial stage of diabetic retinopathy. During this phase, hyperglycemia-induced damage to retinal microvascular endothelial cells (ECs) causes breakdown of the blood-retinal barrier (BRB), increased vascular permeability, and the formation of microaneurysms and hemorrhages before proliferative changes occur (26). Key mechanisms include AGEs binding to RAGE receptors (27), which activates NF-κB and promotes oxidative stress/ROS production (28–30). This decreases nitric oxide (NO) bioavailability (31), elevates adhesion molecules like ICAM-1/VCAM-1 to facilitate leukocyte adhesion (leukostasis) (32), and disrupts tight junctions (such as occludin, ZO-1) through TGF-β and VEGF signaling (33–36). These processes lead to EC apoptosis, basement membrane thickening, and pericyte loss—all of which worsen ischemia and set the stage for progression to vision-threatening complications (37, 38). Its importance lies in its role as the primary trigger of the cycle of inflammation and microvascular rarefaction. Additionally, hyperglycemia increases endothelin-1 (ET-1), a potent vasoconstrictor, which contributes to capillary occlusion and ischemia. Preclinical models have shown that ET-1 receptor antagonists reduce retinal ischemia in diabetic rats, offering a potential therapeutic target (26).

#### 2.1.4 Disruption of the BRB

Disruption of the blood-retinal barrier (BRB) is a key early sign of NPDR, acting as the main functional outcome of earlier microvascular changes like pericyte death and endothelial problems. These changes directly cause plasma leakage, retinal swelling, and the buildup of hard exudates that can interfere with vision, even before proliferative signs appear (26). Advanced imaging studies, such as fluorescein angiography, have revealed that microaneurysms in early NPDR are focal sites of BRB breakdown, with leakage rates correlating with disease severity (27). Furthermore, previous systematic reviews summarized that microvascular alterations in NPDR are more pronounced in T2DM patients with concurrent hypertension, including changes in BRB permeability (e.g., 2–3 times higher FA leakage), which accelerates retinal damage through increased shear stress and VEGF upregulation highlighting the synergistic role of systemic comorbidities in accelerating retinal damage (28–30).

#### 2.1.5 Neurovascular unit dysfunction

Emerging evidence also points to the role of neurovascular unit dysfunction in early DR. The neurovascular unit, comprising endothelial cells, pericytes, astrocytes, and Müller cells, maintains retinal homeostasis. The neurovascular unit uncoupling initiates glial activation, neuronal apoptosis, blood-retinal barrier leakage, and ischemia, shifting the understanding of DR from a solely vascular disorder to a neurovascular one where early NVU impairment predicts functional deficits like reduced contrast sensitivity and progression to vision-threatening complications (31, 32). Hyperglycemia disrupts this unit by inducing Müller cell gliosis and astrocyte dysfunction, further exacerbating microvascular instability. Preclinical research demonstrated that Müller cell-derived VEGF-A contributes to early BRB breakdown, with targeted VEGF inhibition reducing vascular leakage in diabetic mice (33, 34). These findings underscore the interconnectedness of microvascular and glial changes in DR pathogenesis.

### 2.2 Inflammatory pathways

Inflammation is a central driver of early-stage diabetic retinopathy (DR), particularly in non-proliferative diabetic retinopathy (NPDR), where it exacerbates vascular damage and compromises the blood-retinal barrier (BRB). Pro-inflammatory cytokines, such as tumor necrosis factor-alpha (TNF-α), interleukin-6 (IL-6), and vascular endothelial growth factor (VEGF), play pivotal roles in orchestrating retinal inflammation. A previous meta-analysis confirmed elevated retinal IL-6 levels in early NPDR, correlating with vascular leakage (35). These cytokines promote a pro-inflammatory milieu that amplifies retinal damage through multiple pathways. Differences between T1DM and T2DM influence inflammatory profiles (36). In T1DM, the rapid onset of hyperglycemia drives acute inflammatory responses, with higher TNF-α levels observed in early NPDR compared to T2DM. In contrast, T2DM is associated with chronic low-grade inflammation, exacerbated by metabolic syndrome and obesity, which upregulate IL-6 and C-reactive protein (CRP) (37, 38). A recent pilot study using miRNA 3.0 microarrays profiled DR subtypes, revealing subtype-specific inflammatory markers in ocular fluids: vitreous humor showed predominant miRNA upregulation that drives acute cytokine release in T1DM-PDR, while aqueous humor exhibited a unique increase in miR-455-3p in T2DM-NPDR, highlighting T1DM's faster inflammatory response compared to T2DM's more chronic, fluid-specific profile (39). Additionally, a cross-sectional analysis of tear and plasma cytokines in well-controlled T2DM patients without retinopathy found increased IL-6 and TNF-α in tears correlating with systemic low-grade inflammation (40), suggesting tear-based biomarkers could enable early detection of T2DM-related inflammatory changes before NPDR develops.

#### 2.2.1 Leukocyte adhesion

Leukocyte adhesion to the retinal endothelium, mediated by intercellular adhesion molecule-1 (ICAM-1), is a critical mechanism exacerbating BRB disruption. ICAM-1 upregulation, driven by hyperglycemia-induced nuclear factor-kappa B (NF-κB) activation, facilitates leukocyte-endothelial interactions, leading to capillary occlusion and localized ischemia. Previous clinical studies demonstrated that ICAM-1 expression is significantly elevated in the vitreous of patients with early NPDR, correlating with increased retinal vascular permeability (39). Studies also identified elevated levels of monocyte chemoattractant protein-1 (MCP-1), which recruits macrophages to the retina, further amplifying inflammation (40). Additionally, a recent bioinformatics analysis of immune infiltration in human DR tissues identified ICAM-1 and MCP-1 as key hub genes in leukocyte adhesion networks, with ceRNA (lncRNA-miRNA) axes enhancing NF-κB activation in early NPDR, suggesting RNA therapeutics to break the cycle of ischemia-driven inflammation (41).

#### 2.2.2 Inflammasome

The inflammasome particularly the NLRP3, has emerged as a key contributor to retinal inflammation in early DR. Hyperglycemia and oxidative stress activate NLRP3, leading to the release of IL-1β and IL-18, which perpetuate a cycle of inflammation and tissue damage. A previous rodent experiment showed that NLRP3 inflammasome activation in retinal Müller cells drives IL-1β production, contributing to pericyte loss and microvascular dysfunction in diabetic mice (42). Pharmacological inhibition of NLRP3 with MCC950 reduced retinal inflammation and vascular leakage in preclinical models, suggesting a potential therapeutic target (43). Complementing this, a 2024 review on NLRP3 therapeutics in DR highlighted new inhibitors like tonabersat, which block P2X7R-mediated ATP signaling in retinal microglia, decreasing IL-1β/IL-18 release and microglial polarization to hinder progression from early NPDR to proliferative stages (44).

#### 2.2.3 VEGF and complement system

This growth factor traditionally associated with angiogenesis in advanced DR, also plays a significant role in early NPDR by promoting vascular permeability. A clinical study found that vitreous VEGF levels are elevated in early NPDR (45), even in the absence of neovascularization, and correlate with macular edema. The synergistic interaction between VEGF and IL-6 amplifies endothelial dysfunction and BRB breakdown (46). Additionally, the complement system, particularly complement component C3, contributes to retinal inflammation. Increased C3 has been found to be activated in the retina of T2DM patients with NPDR, linking complement dysregulation to leukocyte recruitment and vascular damage (47). Complementing this, a prospective cohort of 252 T2DM patients showed significantly higher serum C3 and C5 levels in NPDR compared with non-DR groups, and a multifactorial analysis confirmed C3 activation as an independent predictor of DR development and progression (48).

#### 2.2.4 Neuroinflammation

Emerging evidence highlights the role of neuroinflammation in early DR, involving retinal glial cells such as microglia and Müller cells. Activated microglia release pro-inflammatory cytokines, including TNF-α and IL-6, which disrupt retinal homeostasis. Microglial activation can precede vascular changes in early NPDR, suggesting that neuroinflammation may be an early therapeutic target (49). Müller cells, which support retinal neurons, also contribute to inflammation by upregulating glial fibrillary acidic protein (GFAP) and VEGF under hyperglycemic conditions (50). Preclinical studies have shown that targeting Müller cell-derived inflammation with anti-inflammatory agents, such as minocycline, reduces retinal cytokine levels and improves BRB integrity (51). A study shows that RIP3 (receptor-interacting protein kinase 3)-mediated necroptosis in retinal microglia triggers the release of pro-inflammatory cytokines such as TNF-α and IL-6, promoting neuroinflammation and early neurodegeneration in diabetic retinopathy (DR) models, with genetic RIP3 knockout significantly reducing microglial activation, cytokine production, and retinal homeostasis disruption (52). Another investigation found that hepatoma-derived growth factor (HDGF), secreted from activated Müller cells, enhances microglial polarization toward a pro-inflammatory M1 phenotype via Dectin-1 signaling, worsening cytokine storms and BRB disruption in early NPDR (53, 54).

### 2.3 Oxidative stress

Oxidative stress is a cornerstone of early-stage diabetic retinopathy (DR) pathogenesis, driven by hyperglycemia-induced overproduction of reactive oxygen species (ROS) that overwhelm the retina's antioxidant defenses. This imbalance leads to lipid peroxidation, protein damage, DNA oxidation, and retinal cell apoptosis, contributing to microvascular and neuronal damage in NPDR (52). Previous research identified mitochondrial dysfunction as a key driver of ROS in early DR, with targeted antioxidants showing promise in preclinical models (53).

Hyperglycemia triggers ROS production through multiple pathways, including the polyol pathway, protein kinase C (PKC) activation, and advanced glycation end product (AGE) formation. In the polyol pathway, excess glucose is metabolized by aldose reductase into sorbitol, depleting NADPH and reducing glutathione levels, a critical antioxidant. It was reported that aldose reductase inhibitors, such as epalrestat, reduced ROS levels and retinal lipid peroxidation in diabetic rats, suggesting a therapeutic avenue (54). PKC activation, particularly the PKC-β isoform, enhances ROS production by upregulating NADPH oxidase, a major source of superoxide in the retina. Elevated PKC-β activity was found in the vitreous of T2DM patients with early NPDR, correlating with increased oxidative damage markers (55).

It is a central contributor to oxidative stress in early DR. Hyperglycemia disrupts mitochondrial electron transport chain function, leading to superoxide overproduction. Previous basic study elucidated that mitochondrial DNA (mtDNA) damage in retinal endothelial cells amplifies ROS production, triggering apoptosis and pericyte loss. In addition, mitochondria-targeted antioxidants, such as MitoQ, reduced retinal ROS levels and preserved BRB integrity in diabetic mice (52). It also found that mitochondrial sirtuin-3 (SIRT3) downregulation in exacerbating oxidative stress, with SIRT3 agonists showing potential to restore mitochondrial function and reduce retinal damage in preclinical models (56).

The interplay between oxidative stress and inflammation amplifies retinal injury (52). ROS activate nuclear factor-kappa B (NF-κB), upregulating pro-inflammatory cytokines like TNF-α and IL-6, which further exacerbate oxidative damage. It was also shown that ROS-induced NF-κB activation in retinal Müller cells promotes VEGF expression, contributing to vascular leakage in early NPDR (57). Similarly, oxidative stress enhances AGE-RAGE signaling, which amplifies ROS production and inflammation. It was demonstrated that elevated retinal AGE levels correlate with oxidative stress markers and NPDR severity in T2DM patients (58). A bibliometric analysis of over 2,500 publications from 2000 to 2024 highlights NLRP3 inflammasome activation and autophagy as emerging hotspots in the pathogenesis of oxidative stress-mediated diabetic retinopathy (DR). Co-citation networks reveal a surge in studies connecting mitochondrial ROS overproduction to early NPDR progression and proposing Nrf2 agonists as new therapeutic agents to restore antioxidant balance (59).

Differences between T1DM and T2DM influence oxidative stress profiles (60). In T1DM, prolonged insulin deficiency may lead to acute ROS surges, with higher levels of lipid peroxidation products like malondialdehyde (MDA) observed in early NPDR (61, 62). In contrast, T2DM may be associated with chronic oxidative stress, exacerbated by dyslipidemia and hypertension, which increase retinal 8-hydroxydeoxyguanosine (8-OHdG), a marker of DNA oxidative damage (63). It is acknowledged that T2DM patients with metabolic syndrome had significant features of oxidative stress (64, 65), which may associate with faster NPDR progression.

Neurovascular unit dysfunction also intersects with oxidative stress (66). Retinal ganglion cells and Müller cells, critical components of the neurovascular unit, are highly susceptible to ROS-induced damage. It was also found that ROS-mediated endoplasmic reticulum (ER) stress in Müller cells upregulates CHOP (C/EBP homologous protein), promoting apoptosis and glial activation in early DR (67, 68).

### 2.4 Advanced glycation end products (AGEs)

Advanced glycation end products (AGEs) are a critical driver of retinal damage in early-stage diabetic retinopathy (DR), particularly in non-proliferative diabetic retinopathy (NPDR) (69). Formed through non-enzymatic glycation of proteins, lipids, and nucleic acids under chronic hyperglycemia, AGEs accumulate in the retina, cross-linking extracellular matrix (ECM) proteins and activating the receptor for AGEs (RAGE). This interaction triggers nuclear factor-kappa B (NF-κB) signaling, amplifying inflammation and oxidative stress, which exacerbate microvascular and neuronal damage (70, 71). Additionally, AGEs modify intracellular proteins, including histones, leading to epigenetic changes that upregulate pro-inflammatory and pro-apoptotic genes (72). Single-cell RNA sequencing study identified that AGE-induced epigenetic modifications in retinal endothelial cells enhance the expression of VEGF and interleukin-6 (IL-6), contributing to BRB breakdown (73). Recent clinical efforts to target the AGE-RAGE axis have produced mixed results but remain very promising (74). Although early RAGE inhibitors like aminoguanidine were discontinued in phase III because of safety issues, newer small-molecule and antibody-based candidates are now in phase II trials for diabetic retinopathy (75, 76).

Differences between T1DM and T2DM influence AGE accumulation and its impact. In T1DM, prolonged hyperglycemia leads to rapid AGE formation, with higher retinal levels of Nε-(carboxymethyl) lysine (CML), a common AGE, observed in early NPDR (74). In T2DM patients, often with dyslipidemia and obesity, exhibit elevated levels of methylglyoxal-derived AGEs, which are linked to chronic inflammation (75).

## 3 T1DM vs. T2DM: distinct pathophysiological profiles

While T1DM and T2DM share core mechanisms, their DR progression differs. T1DM, driven by absolute insulin deficiency, exhibits rapid microvascular damage, with a 5-year incidence of DR approaching 25% in poorly controlled patients (76). In contrast, T2DM is influenced by insulin resistance, metabolic syndrome, and comorbidities like hypertension and dyslipidemia, which accelerate vascular remodeling (77).

### 3.1 Molecular and metabolic distinctions

In T1DM, the abrupt onset of hyperglycemia due to insulin deficiency triggers acute metabolic stress, leading to rapid activation of pathways such as the polyol pathway, protein kinase C (PKC), and advanced glycation end product (AGE) formation. These pathways drive oxidative stress and inflammation, accelerating pericyte loss and blood-retinal barrier (BRB) breakdown. The Diabetes Control and Complications Trial/Epidemiology of Diabetes Interventions and Complications (DCCT/EDIC) study reported that T1DM patients with poor glycemic control (HbA1c >9%) had a 25% incidence of NPDR within 5 years, with elevated retinal levels of Nε-(carboxymethyl) lysine (CML), a key AGE, correlating with microaneurysm formation (74). Additionally, T1DM is associated with higher retinal levels of pro-inflammatory cytokines, particularly tumor necrosis factor-alpha (TNF-α), which amplify endothelial dysfunction.

In T2DM, insulin resistance and associated comorbidities, such as obesity, dyslipidemia, and hypertension, create a chronic low-grade inflammatory state that exacerbates retinal pathology. The metabolic syndrome amplifies AGE-RAGE signaling, with methylglyoxal-derived AGEs being particularly prominent (78). Dyslipidemia in T2DM also contributes to retinal lipid peroxidation, with oxidized low-density lipoprotein (ox-LDL) promoting macrophage infiltration and vascular leakage (79, 80).

### 3.2 Microvascular and inflammatory differences

Microvascular changes in T1DM are characterized by rapid pericyte apoptosis and basement membrane thickening due to intense hyperglycemic stress. It was highlighted that reduced platelet-derived growth factor (PDGF) signaling in T1DM retinas leads to pericyte dropout, destabilizing capillaries and promoting microaneurysms (81). In contrast, T2DM exhibits more pronounced endothelial dysfunction driven by hypertension and dyslipidemia, which synergize with hyperglycemia to increase vascular permeability (82, 83). However, T2DM patients with hypertension may have higher incidence of retinal vascular leakage compared to T1DM patients, mediated by elevated endothelin-1 (ET-1) and vascular cell adhesion molecule-1 (VCAM-1) expression (84, 85).

Inflammatory profiles also differ. T1DM is associated with acute inflammatory responses, with higher retinal levels of interleukin-1β (IL-1β) and TNF-α driving leukocyte adhesion and BRB breakdown. Single-cell RNA sequencing study revealed that T1DM retinas exhibit selective upregulation of IL-1β in retinal microglia, contributing to early neuroinflammation (86). In T2DM, chronic inflammation predominates, with elevated IL-6 and CRP levels linked to systemic comorbidities, correlating with macrophage infiltration and NPDR progression (87, 88).

While direct head-to-head retinal studies remain limited—most compare systemic/serum markers or animal models—emerging evidence from recent research highlights subtle differences: T1DM exhibits a more acute, leukocyte-driven profile with rapid TNF-α surges and NLRP3-dominant inflammasome activation in microglia, linked to sudden hyperglycemia (89); T2DM shows a chronic, low-grade profile amplified by metabolic syndrome, with sustained IL-6 elevation, macrophage infiltration, and complement dysregulation, often correlating with dyslipidemia and hypertension (90). These differences suggest tailored anti-inflammatory strategies, such as NLRP3 inhibitors for T1DM and IL-6 blockers for T2DM (91–93).

### 3.3 Neurovascular and systemic implications

The neurovascular unit, comprising endothelial cells, pericytes, Müller cells, and retinal ganglion cells, is differentially affected. In T1DM, rapid neuronal apoptosis due to oxidative stress and neuroinflammation precedes vascular changes, with retinal ganglion cell loss detected in early NPDR. T1DM patients had a 15% reduction in retinal ganglion cell density within 3 years of NPDR onset, compared to 8% in T2DM patients (89). In T2DM, Müller cell gliosis and glial fibrillary acidic protein (GFAP) upregulation are more pronounced, driven by chronic inflammation and dyslipidemia (90, 91).

Systemically, T2DM patients face a higher burden of comorbidities that exacerbate DR. The Atherosclerosis Risk in Communities (ARIC) study found that T2DM patients with hypertension and dyslipidemia had a higher risk of cardiovascular events, with retinal microvascular changes (e.g., arteriolar narrowing) serving as a predictor of systemic risk (92). In T1DM, patients are more likely to develop other macro/-microvascular complications when predisposed with PDR, with the DCCT/EDIC study reporting a higher incidence of cardiovascular disease in PDR independent of HbA1c level (93).

### 3.4 Diagnostic and therapeutic implications

The distinct pathophysiological profiles of T1DM and T2DM necessitate tailored diagnostic and therapeutic strategies. In T1DM, early screening with optical coherence tomography angiography (OCTA) is critical due to the rapid onset of microvascular changes. It was demonstrated that OCTA detects capillary dropout in T1DM patients 12 months earlier than in T2DM patients, enabling timely intervention (94, 95). In T2DM, integrating systemic risk assessment (e.g., blood pressure, lipid profiles) with retinal imaging enhances risk stratification (96–98). Artificial intelligence (AI)-based algorithms, such as those validated in 2023, shows higher sensitivity for detecting NPDR due to more pronounced microvascular lesions (99, 100). The recent OCTA studies revealed that changes in OCT-A parameters detected in FAZ area and VD could discern the pathological change between type1 and type 2 diabetic patients, along with different stages of DR (101, 102).

Therapeutically, T1DM patients benefit from intensive glycemic control, as demonstrated by the DCCT/EDIC study, which reported a 50% reduction in NPDR progression with tight HbA1c control (< 7%) (DCCT/EDIC, 2023) (103). In T2DM, therapies targeting systemic comorbidities, such as SGLT-2 inhibitors and GLP-1 receptor agonists, are critical. The clinical trials have shown that SGLT-2i reduced NPDR progression in T2DM patients with hypertension, likely due to its anti-inflammatory and hemodynamic effects (104, 105). Although there is no clear relationship between specific GLP-RA Semaglutide and the risk for retinopathy according to the SUSTAIN-6 and a series clinical observations (possibly due to the inappropriate administrative manner), however, the other GLP-1Ras did not receive these reports, and real-world evidence further supports the use of GLP-1 agonists could potentially reduce DR risk and progression (12, 106). In summary, T1DM and T2DM exhibit distinct pathophysiological profiles in early DR, with T1DM driven by acute hyperglycemic stress and rapid microvascular changes, and T2DM characterized by chronic inflammation and systemic comorbidities. These differences influence the molecular, microvascular, and neurovascular features of NPDR, necessitating tailored diagnostic and therapeutic approaches. Recent studies and clinical trials underscore the importance of personalized medicine to address these unique profiles and optimize outcomes in early DR.

## 4 Diagnostic advances: precision detection and systemic risk prediction

**Figure 2:**
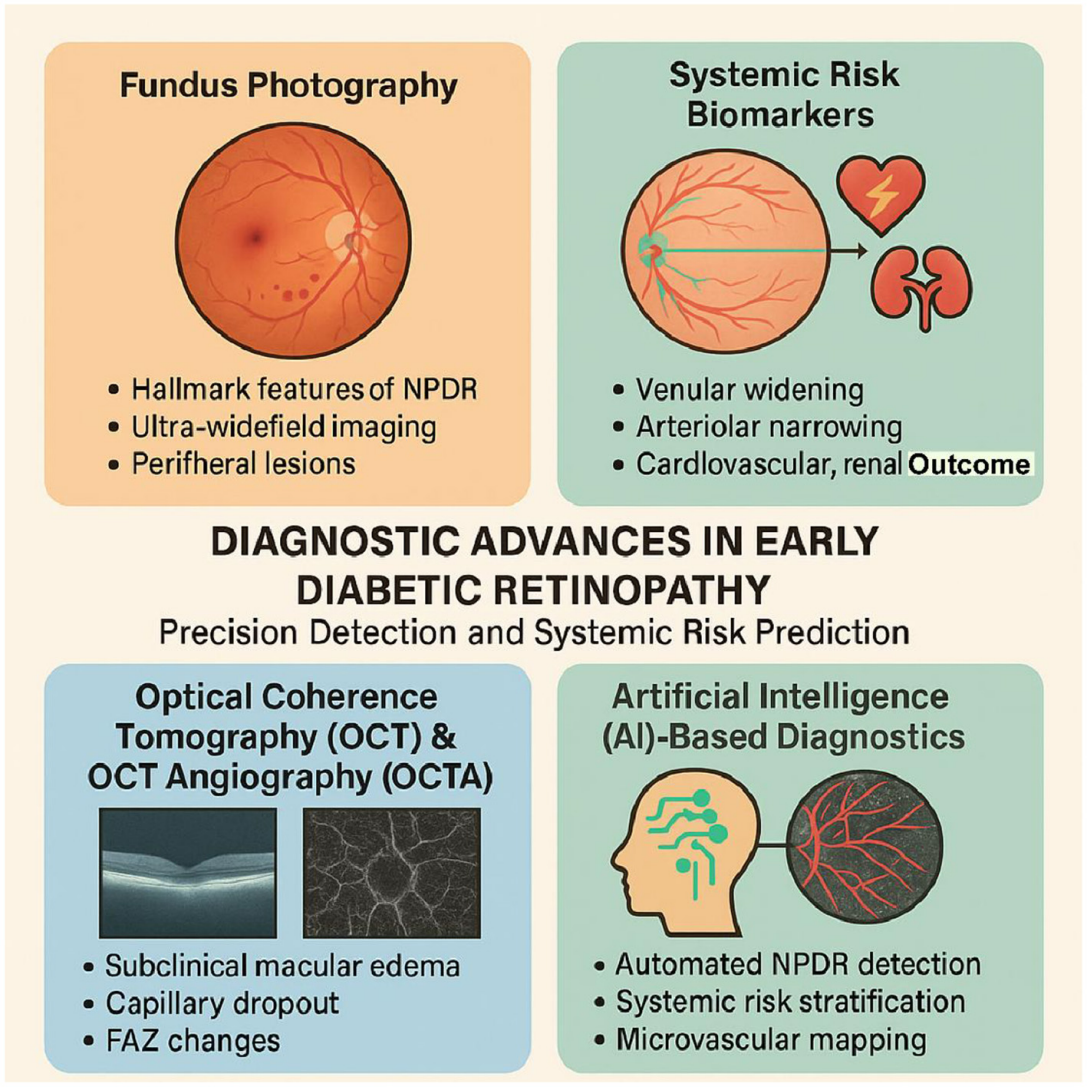
Diagnostic Advances in Early Diabetic Retinopathy key advancements in diagnostic technologies for early detection and risk assessment in diabetic retinopathy (DR). Fundus Photography Traditional and ultra-widefield imaging for capturing microaneurysms and peripheral lesions in NPDR. Optical Coherence Tomography (OCT) & OCT Angiography (OCTA) High-resolution imaging revealing subclinical macular edema, capillary dropout, and FAZ changes, with OCTA offering quantifiable metrics like vessel density for prognostic use. Artificial Intelligence (AI)-Based Diagnostics FDA-approved and deep learning models enable automated NPDR detection, systemic risk stratification, and microvascular mapping with high sensitivity and specificity. Systemic Risk Biomarkers – Retinal vascular features (e.g., venular widening, arteriolar narrowing) predict cardiovascular and renal outcomes in diabetic patients, especially in T2DM.

Retinal vascular changes serve as biomarkers for systemic complications, such as cardiovascular disease and CKD, offering a non-invasive window into diabetes-related comorbidities (8). This section explores the latest developments in fundus photography, optical coherence tomography (OCT) and OCT angiography (OCTA), AI-based diagnostics, and systemic risk biomarkers, supported by recent clinical and real-world evidence (Figure 2).

### 4.1 Fundus photography

Fundus photography remains the cornerstone of DR screening, providing a non-invasive method to detect hallmark features of early NPDR, including microaneurysms, hemorrhages, and hard exudates. Its widespread availability and cost-effectiveness make it ideal for population-based screening programs. Automated systems, such as those using ultra-widefield (UWF) fundus photography, expand the field of view, capturing peripheral retinal lesions (107–109). UWF fundus photography was demonstrated to detect 15% more peripheral microaneurysms in T2DM patients compared to standard fundus photography, improving early NPDR diagnosis (110).

### 4.2 Optical coherence tomography (OCT) and OCT angiography (OCTA)

Optical coherence tomography (OCT) provides high-resolution, cross-sectional imaging of the retina, enabling precise quantification of retinal thickness and detection of subclinical macular edema, a common complication in early NPDR (111). Spectral-domain OCT (SD-OCT) and swept-source OCT (SS-OCT) offer micrometer-level resolution, allowing visualization of retinal layer alterations before clinical symptoms emerge (112, 113). Recent study demonstrated that SD-OCT detected retinal nerve fiber layer (RNFL) thinning in 20% of T1DM patients with no visible NPDR, indicating early neurodegeneration (114). OCT is also critical for monitoring treatment response, with changes in central subfield thickness serving as a key endpoint in clinical trials (115, 116).

OCT angiography (OCTA), a non-invasive technique, visualizes retinal and choroidal vasculature without the need for contrast agents, identifying capillary dropout, non-perfusion areas, and microvascular remodeling in early NPDR (117). OCTA's ability to detect microvascular changes earlier than fundus photography was validated in a longitudinal study, which showed that OCTA identified capillary dropout in 25% of T2DM patients with normal fundus photography findings (118, 119). These studies also highlighted OCTA's sensitivity in detecting foveal avascular zone (FAZ) enlargement, a marker of early ischemia, which correlated with a higher risk of NPDR progression (120). Furthermore, OCTA's ability to quantify vessel density and perfusion metrics enhances its prognostic value (121). In recent studies, reduced deep capillary plexus density on OCTA could predict an increased risk of NPDR worsening in T2DM patients (122, 123). However, challenges such as motion artifacts and limited field of view are being addressed with widefield OCTA systems. Recent trial demonstrated that widefield OCTA detected peripheral non-perfusion in patients with early NPDR, improving risk stratification, as well as identify central and peripheral neurovascular and microstructural impairments in patients with full-course DR (124, 125). Multiple trials are also evaluating OCTA's utility in predicting systemic complications, with preliminary data suggesting a correlation between FAZ enlargement and cardiovascular risk (126, 127).

### 4.3 Artificial intelligence (AI)-based diagnostics

AI-based algorithms, trained on large datasets of retinal images, have transformed DR diagnosis by achieving over 95% accuracy in detecting NPDR and predicting its progression. The FDA-approved IDx-DR system, validated in 2023, integrates seamlessly into primary care settings, enhancing access to screening for underserved populations. This system, which analyzes fundus photographs for microaneurysms and hemorrhages, achieved 95% sensitivity and 91% specificity in pooled analyses in a systematic review (128). Deep learning models, such as convolutional neural networks (CNNs), further improve diagnostic precision by identifying subtle features (129), such as intraretinal microvascular abnormalities (IRMAs), that may be missed by human graders (130, 131).

AI's predictive capabilities extend beyond DR detection to systemic risk assessment. In recent study, linked retinal venular widening, detected by AI-analyzed fundus images, to an increased risk of cardiovascular events in T2DM patients, highlighting the retina's role as a systemic biomarker (132). Similarly, AI-based analysis of OCTA images can predict NPDR progression by quantifying vessel density and FAZ metrics (133). A 2025 study reported that AI models trained on OCTA data predicted NPDR worsening in T2DM patients, outperforming traditional grading methods (134).

### 4.4 Systemic risk biomarkers

Retinal vascular changes, such as arteriolar narrowing, venular dilation, and tortuosity, serve as biomarkers for systemic complications, including hypertension, stroke, and CKD (135). The Atherosclerosis Risk in Communities (ARIC) study reported that retinal microvascular abnormalities, detected via fundus photography, predicted a higher risk of heart failure in diabetic patients, with stronger associations in T2DM due to its systemic comorbidities (136). Similarly, other previous studies found that retinal venular widening correlated with an increased risk of CKD progression in T2DM patients, highlighting the retina's predictive value (137, 138). Emerging biomarkers include retinal vessel caliber, fractal dimension, and tortuosity, quantifiable through AI and OCTA. Clinical trials are exploring the prognostic utility of retinal biomarkers. These advancements underscore the retina's role as a non-invasive window into systemic health, facilitating interdisciplinary management of diabetes-related complications.

In summary, diagnostic advances in fundus photography, OCT/OCTA, AI-based diagnostics, and systemic risk biomarkers have transformed early DR detection and risk prediction. Fundus photography remains a scalable screening tool, while OCT and OCTA offer high-resolution insights into retinal and microvascular changes. AI enhances diagnostic accuracy and accessibility, and retinal biomarkers provide prognostic value for systemic complications. These tools, supported by clinical trials and real-world evidence, enable personalized approaches to managing early NPDR and its systemic implications.

### 4.5 From neurosensory dysfunction to visible microvascular lesions: the role of functional diabetic retinopathy

It is now widely accepted that the earliest insult to the diabetic retina is neurodegenerative rather than vasculopathy (139, 140). Long before microaneurysms, dot-blot hemorrhages, or hard exudates become visible on fundus photographs or ophthalmoscopy, the retina has already sustained measurable neuronal and synaptic loss, reactive gliosis, impaired neurovascular coupling, and subtle but objective deficits in contrast sensitivity, color discrimination, dark adaptation, and electrophysiological responses. This preclinical, pre-vascular phase—during which the fundus still appears essentially normal under current clinical grading systems—has come to be recognized as functional diabetic retinopathy.

The implications are straightforward yet profound. Conventional screening programmers and clinical trial endpoints remain almost entirely anchored to the detection of visible microvascular abnormalities and to changes in foveal visual acuity. Because foveal acuity reflects the function of less than 1 % of the retinal surface and remains remarkably preserved until relatively late in the disease, these traditional metrics are inherently blind to the diffuse neurosensory disturbance that characterizes the earliest stages of diabetic retinal disease. Patients who are reassuringly labeled “no apparent retinopathy” or “mild non-proliferative retinopathy” on seven-field photography frequently already harbor substantial, widespread, and potentially reversible functional damage when examined with wider-field or multifocal techniques.

Functional diabetic retinopathy therefore represents the true initial stage of the condition—a stage in which the retina is no longer normal, even though it still looks normal by yesterday's standards.

## 5 Therapeutic strategies: current and emerging interventions

The management of early-stage diabetic retinopathy (DR), particularly non-proliferative diabetic retinopathy (NPDR), focuses on halting disease progression through glycemic control, lifestyle interventions, and targeted pharmacotherapies. These strategies aim to address the underlying pathophysiological mechanisms—microvascular damage, inflammation, oxidative stress, and advanced glycation end product (AGE) accumulation—while mitigating systemic complications.

### 5.1 RAAS inhibitors and MRA

Renin-angiotensin-aldosterone system (RAAS) inhibitors, including angiotensin-converting enzyme (ACE) inhibitors and angiotensin II receptor blockers (ARBs), reduce retinal vascular damage by lowering systemic blood pressure and local inflammation, thereby stabilizing the BRB (141). Finerenone, a nonsteroidal mineralocorticoid receptor antagonist, has emerged as a promising agent due to its anti-inflammatory and anti-fibrotic effects. The FIDELITY analysis (2024) of the FIDELIO-DKD and FIGARO-DKD trials reported a 19% reduction in combined kidney and cardiovascular outcomes in T2DM patients, with a lower incidence of DR progression in the finerenone group compared to placebo (11). Basic studies demonstrated that finerenone may lower VEGF, ICAM-1, and IL-1ß. In oxygen-induced retinopathy, it reduced neovascularization, vascular leakage, and microglial density while increasing Tregs in the blood, spleen, and retina (142).

### 5.2 GLP-1 receptor agonists

GLP-1 receptor agonists, such as liraglutide and semaglutide, improve glycemic control while providing retinal protection through anti-inflammatory and anti-apoptotic mechanisms (143). Although they have achieved unprecedented success in preventing macrovascular complications in multiple CVOTs trials, the increased risk of DR progression raises concerns about potential harm to patients' vision receiving this treatment (144). Contrary to the beneficial findings in basic studies, where GLP-1 analogs could alleviate metabolic lesions in the retina's parenchyma or vessel bed and demonstrate neural protective effects, the discrepancy between preclinical results and real-world practice has sparked safety concerns about expanding their use. Many criticisms suggest that this adverse outcome may be directly linked to the rapid normalization of HbA1c or blood glucose levels through individualized dosing, leading to early worsening of vision, especially in patients with diabetic retinopathy. This effect is also observed with DPP-IV inhibitors (145) and in recent reports involving Tirzepatide, a single molecule that combines GLP-1R and GIPR dual agonists (146). Updated systematic reviews further support the idea that GLP-1 RA administration does not inherently increase DR risk through specific class effects of incretin hormones but rather reflects a common phenomenon of rapid hypoglycemia correction from severe hyperglycemia (143, 147–149). To promote effective and safe treatment outcomes, clinical guidelines increasingly emphasize the use of these medications, supported by real-world evidence and monitoring for adverse effects (150). Despite reports of non-arteritic anterior ischemic optic neuropathy (NAION), ongoing trials and advances in basic research are expected to find better solutions or strategies that harness the benefits of GLP-1 RAs while minimizing concerns about side effects previously (151, 152).

### 5.3 SGLT-2 inhibitors

SGLT-2 inhibitors, such as empagliflozin and dapagliflozin, lower blood glucose by promoting urinary glucose excretion and reduce cardiovascular and renal risks (153, 154), thereby indirectly benefiting retinal health. Both RCTs and real-world evidence have demonstrated that SGLT-2 inhibitors slow the progression of NPDR in T2DM patients (135, 155). These inhibitors also appear to benefit other microvascular systems, including diabetic dementia (156, 157), neurodegenerative diseases (156, 157), cardiomyopathy (158, 159), and pulmonary dysfunction (160), likely through mechanisms that enhance endothelial function and exert anti-inflammatory effects (161–164). Numerous basic studies have further clarified metabolic and vascular protective mechanisms beyond those observed in clinical and population-based studies (16, 165–168). In T1DM, SGLT-2 inhibitors are used cautiously due to the risk of diabetic ketoacidosis. Similar to the therapeutic approach of GLP-1RA in T1D demonstrated in multiple clinical trials (169–171), microvascular protection might also be achieved with SGLT-2 inhibitors, with some trials aiming to minimize the risk of ketosis (172), such as by using glucagon (GCG) inhibitors (173–176), as research has shown specific benefits that could be derived from this, independent of the different pathophysiology of microvascular complications in T2DM (177).

### 5.4 GIP analogs and glucagon-based therapies

Dual glucose-dependent insulinotropic polypeptide (GIP)/GLP-1 receptor agonists, such as tirzepatide, provide synergistic benefits for glycemic control, weight loss, and systemic inflammation, making them promising for early DR management. The SURPASS trials showed that tirzepatide achieved significant HbA1c reductions and was not linked to NPDR progression compared to placebo (178). Glucagon (GCG)-based therapies, which boost energy expenditure and decrease adiposity (179), have been developed. These include GLP-1R/GCGR co-agonists and GLP-1R/GIPR/GCGR tri-agonists, which have demonstrated exceptional effects on weight loss and metabolic enhancement (180, 181). Preclinical studies have already explored the relationship between GCGR and microvascular systems. In mice with GCGR knockout, there may be a delayed loss of retinal function, decreased visual acuity, and eventual retinal cell death (182). GCG could play a protective role in the nervous system, showing protective effects through GCGR activation (183). Although evidence of GCG's role in NPDR is limited, substantial basic and clinical evidence exists regarding GCG/GCGR's impact on the renal microvascular system. Elevated GCG levels have been associated with changes in eGFR and sodium-fluid retention. Hyperglucagonemia after OGTT has been linked to increased UACR and glomerular injury, as confirmed by multiple preclinical studies (184–187). However, the main issue appears to be how to reduce levels of both GCG and insulin. Since GCG is not a primary hormone responsible for diabetic kidney disease (DKD), it may serve as a key contributor to renal pathophysiology. This explains the paradox of the current clinical success observed in the FLOW trial of semaglutide (188, 189), while also accounting for its limited use in certain populations at higher risk of DR. Future research should focus on not only optimizing the GCG/insulin ratio for microvascular protection but also uncovering the core pathophysiological mechanisms linked to DR. Additionally, we expect updates on the mechanisms driving renal or other microvascular outcomes related to GLP-1/GIP or GLP-1/GCG trials.

### 5.5 The effect of adjusting lipid metabolism and beyond

Fenofibrate, originally a lipid-lowering drug, notably slows the progression of diabetic retinopathy (DR) and decreases the need for laser treatment in type 2 diabetes, as demonstrated by two large clinical trials (190–192). The first laser treatment was less often required in the fenofibrate group (3.4% vs. 4.9% in the placebo group; HR 0.69, 95% CI 0.56–0.84; *p* = 0.0002), with an absolute risk reduction of 1.5%. In patients with existing DR, fenofibrate reduced the progression of 2-step retinopathy (3.1% vs. 14.6%; *p* = 0.004) and a composite endpoint that included progression, macular edema, or laser treatments (HR 0.66, 95% CI 0.47–0.94; *p* = 0.022). These benefits appear to be independent of lipid levels, indicating that broader mechanisms may be involved (193).

Preclinical studies show fenofibrate's protective effects on retinal cells (194, 195). Its active metabolite, fenofibric acid (FA), prevents apoptosis of retinal endothelial cells and maintains the blood-retinal barrier by preventing tight junction disorganization and hyperpermeability in diabetic conditions. FA also downregulates basement membrane components like fibronectin and collagen IV, reducing permeability, and promotes autophagy and survival pathways in retinal pigment epithelium cells. Fenofibrate decreases retinal vascular leakage and leukostasis in type 1 diabetic mouse models and inhibits neovascularization in oxygen-induced retinopathy models. It also prevents retinal neurodegeneration in db/db mice, a model for type 2 diabetes, and demonstrates therapeutic effects on diabetic peripheral neuropathy. These actions involve gene regulation impacting inflammation, angiogenesis, and apoptosis, which are key factors in DR development. Fenofibrate's efficacy makes it a promising adjunct therapy for DR management alongside gly cemic and blood pressure control. However, caution is necessary due to potential liver and kidney toxicities. Further trials are needed to confirm its benefits in type 1 diabetes and diverse Asian populations with dyslipidemia.

### 5.6 Future directions

Early-stage diabetic retinopathy (DR) offers a critical opportunity to prevent vision loss in type 1 and type 2 diabetes. Driven by microvascular damage, inflammation, oxidative stress, and advanced glycation end products, DR progresses differently in T1DM (rapid onset) and T2DM (chronic inflammation). Advanced diagnostics like fundus photography, OCT, OCTA, and AI enable early detection and predict systemic risks, such as cardiovascular disease. Therapies, including finerenone, incretin-based therapy, SGLT-2 inhibitors, and emerging neuroprotective treatments may met the need for reducing NPDR progression. Combination therapies, integrating RAAS modulators, GLP-1 agonists, SGLT-2 inhibitors, holding promise for synergistic effects, could exert their potential specific protective ability unless the underlying mechanism and setbacks could be thoroughly comprehended. The integration of these therapies with AI-driven diagnostics could enable personalized treatment plans, optimizing outcomes in early DR. Supported by robust clinical trial data and real-world evidence, these approaches address the multifaceted nature of NPDR, offering hope for preventing vision loss and systemic complications in T1DM and T2DM patients. Future efforts should focus on personalized medicine and accessible solutions to reduce the global DR burden, preserving vision and enhancing patient quality of life.
